# Dimensional comparability of psychosocial working conditions as covered in European monitoring questionnaires

**DOI:** 10.1186/1471-2458-14-1251

**Published:** 2014-12-09

**Authors:** Maren Formazin, Hermann Burr, Cecilie Aagestad, Tore Tynes, Sannie Vester Thorsen, Merja Perkio-Makela, Clara Isabel Díaz Aramburu, Francisco Javier Pinilla García, Luz Galiana Blanco, Greet Vermeylen, Agnes Parent-Thirion, Wendela Hooftman, Irene Houtman

**Affiliations:** Department “Work & Health”, Bundesanstalt für Arbeitsschutz und Arbeitsmedizin (Federal Institute for Occupational Safety & Health), Nöldnerstraße 40-42, 10317 Berlin, Germany; Statens arbeidsmiljøinstitutt (National Institute of Occupational Health), Gydas vei 8, Majorstuen, Oslo, Norway; Det Nationale Forskningscenter for Arbejdsmiljø (National Research Center for the Working Environment), Lersø Parkallé 105, 2100 København Ø, Denmark; Työterveyslaitos (Finnish Institute of Occupational Health), Topeliuksenkatu 41 a A, 00250 Helsinki, Finland; Instituto Nacional de Seguridad e Higiene en el Trabajo (National Institute of Safety and Hygiene at Work), Calle de Torrelaguna 73, 28027 Madrid, Spain; European Foundation for the Improvement of Living and Working Conditions (Eurofound), Wyattville Road, Loughlinstown, Dublin 18, Ireland; TNO, Postbus 3005, 2301, DA Leiden, Netherlands

**Keywords:** Psychosocial working conditions, Monitoring, Surveillance, Job demand-control-support model, Effort-reward-imbalance model, Dimension

## Abstract

**Background:**

In most countries in the EU, national surveys are used to monitor working conditions and health. Since the development processes behind the various surveys are not necessarily theoretical, but certainly practical and political, the extent of similarity among the dimensions covered in these surveys has been unclear. Another interesting question is whether prominent models from scientific research on work and health are present in the surveys – bearing in mind that the primary focus of these surveys is on monitoring status and trends, not on mapping scientific models. Moreover, it is relevant to know which other scales and concepts not stemming from these models have been included in the surveys. The purpose of this paper is to determine (1) the similarity of dimensions covered in the surveys included and (2) the congruence of dimensions of scientific research and of dimensions present in the monitoring systems.

**Method:**

Items from surveys representing six European countries and one European wide survey were classified into the dimensions they cover, using a taxonomy agreed upon among all involved partners from the six countries.

**Results:**

The classification reveals that there is a large overlap of dimensions, albeit not in the formulation of items, covered in the seven surveys. Among the available items, the two prominent work-stress-models – job-demand-control-support-model (DCS) and effort-reward-imbalance-model (ERI) – are covered in most surveys even though this has not been the primary aim in the compilation of these surveys. In addition, a large variety of items included in the surveillance systems are not part of these models and are – at least partly – used in nearly all surveys. These additional items reflect concepts such as "restructuring", "meaning of work", "emotional demands" and "offensive behaviour/violence & harassment".

**Conclusions:**

The overlap of the dimensions being covered in the various questionnaires indicates that the interests of the parties deciding on the questionnaires in the different countries overlap. The large number of dimensions measured in the questionnaires and not being part of the DCS and ERI models is striking. These "new" dimensions could inspire the research community to further investigate their possible health and labour market effects.

**Electronic supplementary material:**

The online version of this article (doi:10.1186/1471-2458-14-1251) contains supplementary material, which is available to authorized users.

## Background

The working conditions of different European countries are monitored by national and EU wide surveys. National as well as European politicians use the results from these surveys for their decision making on issues regarding working conditions and work-related outcomes. In this paper, two issues regarding the monitoring of working conditions at the national level and the EU level are investigated. First, the dimensions of the psychosocial working environment monitored in these surveys and the degree of European consensus are examined. Second, the extent of overlap between these dimensions and dimensions of the most prominent psychosocial models on work and health is investigated.

### Monitoring of working conditions

Surveys on working conditions aim at studying status and trends in working conditions and at identifying risk groups. Surveillance of the work environment needs to include assessments being both cross-sectional – in order to point out vulnerable groups – and longitudinal – in order to allow the follow-up of trends [1]. A necessary prerequisite for a meaningful interpretation of the data is the usage of exactly the same items in each wave of the survey. For consecutive surveys it is always a dilemma to either improve an item and the fact that a change in questioning hinders trend analysis. In addition to following trends, surveys are aimed at grasping new, emerging issues. Hence, the purpose of national surveys is more descriptive than aetiological.

In recent decades of monitoring working conditions, psychosocial working conditions have gained increasing attention in these surveys [2], reflecting the changing world of work and the concern for potential adverse health outcomes caused by psychosocial hazards. The latter are defined as "those aspects of work design and the organisation and management of work, and their social and environment contexts, which have the potential for causing psychological or physical harm" ( [3], in: [4], p.129 f.). Indeed, psychosocial working conditions constitute a large part of these surveys [5, 6]. The importance of this issue in politics is reflected in recommendations by the European Agency for Safety and Health at Work (EU-OSHA, [7]).

In the development of these monitors, there has been little coordination between the countries because these large national surveys are managed and financed by different national institutions, e. g. ministries and statistical bureaus, and other parties such as the social partners (Employers’ and employees’ organizations). Hence, the aim of the different surveys with regard to possible occupational safety and health (OSH) risks and risk groups has been defined in national contexts. E.g. in Denmark, monitoring is carried out by the National Research Centre for the Working Environment on behalf of the Danish Working Environment Authority, and the content of the surveillance programme is negotiated with the social partners. In Germany, monitoring is carried out by the Federal Institute for Occupational Safety and Health (BAuA) together with the Federal Institute for Vocational Education and Training (BIBB).

As a consequence, the content of the questionnaires in each of these surveys is specific to the different countries [6]. Additionally, even if the same concepts are measured, specific concepts might be measured in different ways, making comparisons across countries difficult. In order to derive valid conclusions from comparisons, measurements must be identical [8].

### Psychosocial working conditions & health

The last decades have seen increasing efforts in research on associations between psychosocial working conditions and different health outcomes: Poor working conditions have been shown to be related to incident heart disease [9–11], development of depressive symptoms [12–14], new cases of long term sickness absence [15–17] and disability pensioning [18].

Research on the impact of work on health is – to a large extent – based on two models, namely the demand-control-support model (DCS; [19, 20]) and the effort-reward-imbalance model (ERI; [21, 22]). In several reviews and meta-analyses, these dimensions have been related to health outcomes such as cardiovascular diseases [10, 11, 23], musculoskeletal diseases [24] and mental disorders [12–14, 25]. Nonetheless, recent research has indicated that further psychosocial concepts beyond DCS and ERI seem to be linked to health outcomes [26–28].

Based on our considerations regarding monitoring of working conditions in Europe and on the associations of psychosocial working conditions and health outcomes, we wish to connect scientific research with practical realization on two issues in this paper. First, we explore which dimensions of the psychosocial working environment are monitored in the surveys in Europe. This comparison will point to commonalities as well as to topics measured only in a few national surveys and allow us to make inferences on the comparability across countries. Second, we investigate if the DCS and the ERI, i.e. the most prominent models on work and health in the scientific literature, are covered and to which extent dimensions outside these models with a possible relation to health as has been shown in some studies are covered.

## Methods

### Data

In 2009, six member countries of the "Partnership for European Research in Occupational Safety and Health (PEROSH)", a joint collaboration of European institutes on research and development in occupational safety and health, formed a project group on "Survey development and cross culture methodology": Spain, the Netherlands, Finland, Norway, Denmark and Germany. The group chose questionnaires used in the recurrent surveillance systems that – among other aspects – focus on working conditions and health in their respective countries. Additionally, Eurofound, the European Foundation for the Improvement of Living and Working Conditions whose role is to provide knowledge in the area of social and work-related policies, was invited to join the group as an associate partner. For each country, the national survey focusing on working conditions and health was used as input data. In effect, the following surveys were included: Spanish National Working Conditions survey (ENCT), National Working Condition Survey (NEA) in the Netherlands, Finnish National work and health survey (FNWHS), Norway - Survey of living conditions - Working environment (LKU), Danish Work Environment Cohort Study (DWECS ), German Labour Force Survey (BIBB/BAuA) and European Working Conditions Survey (EWCS). All included surveys are repeated periodically. For each survey, the latest wave with an authorized English translation was selected. In some countries, e. g. Denmark, there has been a new wave with a revised questionnaire; however, it is not available in English as of yet. The items and questions were translated from their original language into English by authorized translators in each country. An exception was Germany where only the older version of the survey from 2006 was translated by professional translators and the items that were new to the latest survey were translated by two English-speaking scientists from the field of occupational safety and health.

### Analysis

The cooperating partners agreed on a taxonomy to classify the psychosocial items of all questionnaires by the following procedure: In a first step, this taxonomy was based on the dimensions of the DCS-model [19, 20] and the ERI-model [21, 22]. As an enhancement to these models in step two, the COPSOQ taxonomy [29] was added to cover a wider field. The COPSOQ is a comprehensive questionnaire based on seven theories in occupational health psychology covering a large variety of dimensions describing psychosocial working conditions [29]. Thus, these dimensions do not originate from COPSOQ, a number of dimensions can also be found in other broad instruments like the QPS Nordic [30]. In step three, we added new dimensions based on other specific concepts because it became evident that the dimensions included so far did not suffice in order to classify all items. In effect, the taxonomy contains 34 dimensions. Table 1 gives an overview from which model (DCS and/or ERI) the dimensions originate and/or whether they are part of COPSOQ. A full table containing all items assessing psychosocial working conditions that have been used in the surveys and that were sorted into the 34 dimension can be found in Additional file 1. All dimensions were sorted into one of six domains (demands at work, work organization and job contents, interpersonal relations and leadership, work-individual interface, values at workplace, offensive behaviours; [29]).

**Table 1:** **Dimensions present in the participating surveys - number of surveys and questionnaires/models of origin**

Domain	Dimensions	No. of surveys in which dimension is measured	Dimension is part of DCS model	Dimension is part of ERI model	Dimension is part of COPSOQ I or II	Other dimensions
**Demands at work**	Demands - general (work load)	4	√			
	Quantitative demands - work pace	7	√	√	√	
	Quantitative demands - amount	7*	√	√	√	
	Emotional demands	7			√	
	Demands on hiding emotions	2			√	
	Cognitive demands	5		√**	√	
	Sensorial demands	2			√	
	Contact with clients, suppliers etc.	5				√
	Interruptions	4		√		
**Work organization and job contents**	Degrees of freedom	7			√	
	Influence/decision authority	7	√		√	
	Possibilities for development/skill discretion	7	√		√	
	Variation/repetition	6	√		√	
	Meaning of work/commitment to the workplace	6			√	
	Organizational influence/org. decision latitude	4				√
	Workplace innovation	3				√
**Interpersonal relations and leadership**	Contact with supervisor/social support by manager	7	√	√	√	
	Contact with co-workers/social support by co-workers	7	√	√	√	
	Teamwork/social community at work	6			√	
	Quality of leadership	3			√	
	Predictability	3			√	
	Recognition	2		√	√	
	Career development	5		√	√	
	Role conflict	3	√		√	
	Role clarity	4			√	
	Financial rewards	5		√	√	
	Safety culture	4				√
	Restructuring	6				√
**Work-individual interface**	Job security	7		√	√	
	Work-life balance	7			√	
**Values at workplace**	Trust	1			√	
	Justice/discrimination	6		√	√	
**Offensive behaviour**	Offensive behaviour/violence & harassment	6			√	
	Workplace conflict	2			√	

*In Finland, the dimension "quantitative demands – amount" is assessed by asking participants about the number of compensated and non-compensated overtime at work.

**The aspect of "having responsibility" – which is part of "cognitive demands" – is assessed in two countries: the European EWCS and the German BIBB-BAuA.

The dimension "reward" that stems from the ERI-model combines different aspects: i) money/promotion, ii) esteem, iii) status control/job security [21, 22]. In other theories and questionnaires, these three aspects are separate constructs. In order to enhance clarity, we have chosen to follow this separation. Consequently, our overview does not contain a combined dimension "reward" but separate aspects as e.g. "financial rewards", "career development" and "job security".

### Ethical approval

Because we analysed the content of questionnaires (and not participant data) ethical approval was not necessary.

## Results

Table 1 gives an overview of the dimensions of psychosocial working conditions used in the seven surveys. A more thorough overview of all dimensions and all items can be found in Additional file 1. 16 out of 34 dimensions (47%) are used in seven or six of the surveys. Additionally, 9 out of 34 dimensions (26%) are used in either four or five of the surveys.

Some domains are covered by most surveys, e.g. "demands at work", "work organization and job contents", and "work-individual interface". In the domain "demands at work", all seven surveys cover the dimensions "emotional demands" and "quantitative demands", the latter with both "work pace" and "amount" items. In the domain "work organization and job contents", the dimensions, "degrees of freedom", "influence/decision authority", "possibilities for development/skill discretion" and "variation/repetition" are covered in 6 or 7 surveys; and in the domain of "work-individual interface", both dimensions "job security" and "work-life balance" are covered in all seven surveys.

An example of a domain that is more sparsely covered is the domain of "interpersonal relations and leadership" where only a minority of dimensions are covered in all seven surveys ("contact with supervisor/social support by supervisor", "contact with co-workers/social support by co-workers") or in six surveys ("social community at work", "restructuring"). The majority of dimensions from this domain are only covered in a two or three surveys, e. g. "role conflict", "recognition" and "predictability".

Even though there is great similarity in which dimensions are covered by the individual surveys one can find a lot of differences in the wording of questions as well as answering schemes in the items assessing the dimensions. For the former, one example are the items assessing "emotional demands": In Denmark, workers are asked whether their work is emotionally draining ("Is your work emotionally draining?" [29]), whereas Norwegian workers are asked how often strong emotional feelings arise at work ("In your work, to what extent do you need to deal with strong feelings such as sorrow, anger, desperation, frustration and so on from customers, clients or other people who are not employed at your workplace?" [26]). The former item includes the workers’ evaluation of their work, whereas the latter item focuses on a description of a situation.

Similarly, answering schemes between countries partly differ. In the German survey, workers are not only asked whether certain work conditions apply to them, but also, whether this is distressing for them. One such example is the assessment of "quantitative demands – work pace": "How frequently does it occur in your work that you have to work very fast? Is that stressful for you?" [31]. This second part of the question is not used in any other country: e. g. in Spain, workers are solely asked "To what extent does your work imply working very fast?" [32].

Tables 2 and 3 give an overview on the degree to which the dimensions of the two prominent models on the psychosocial work environment, i.e. the DCS and the ERI model, are covered in the seven surveys. According to the taxonomy used for classifying items, two or three dimensions make up each of the dimensions from the models. E.g. "control" from the DCS model includes three aspects that are separate dimensions in the taxonomy: "influence/decision authority", "possibilities for development/skill discretion" and "variation/repetition". Similar distinctions hold true for the other dimensions "demand", "support", "effort" and "reward".

**Table 2:** **The DCS model in the surveys**

Dimension of DCS model	Dimension in overview	NL	DK	NO	FI	ES	DE	EU
**Demand**	Quantitative demands - work pace	**√**	**√**	**√**	**√**	**√**	**√**	**√**
	Quantitative demands - amount	**√**	**√**	**√**	√*	**√**	**√**	**√**
	Role conflict	-	**√**	**√**	-	-	-	**√**
**Control**	Influence/decision authority	**√**	**√**	**√**	**√**	**√**	**√**	**√**
	Possibilities for development/skill discretion	**√**	**√**	**√**	**√**	**√**	**√**	**√**
	Variation/repetition	**√**	**√**	**√**	-	**√**	**√**	**√**
**Support**	Contact with supervisor/social support by supervisor	**√**	**√**	**√**	**√**	**√**	**√**	**√**
	Contact with co-workers/social support by co-workers	**√**	**√**	**√**	**√**	**√**	**√**	**√**

*Notes*: √ = dimension is available in the survey; - = dimension is not available in the survey; *In Finland, the dimension "quantitative demands – amount" is assessed by asking participants about the number of compensated and non-compensated overtime at work.

NL = Netherlands; DK = Denmark; NO = Norway; FI = Finland; ES = Spain; DE = Germany; EU = Europe.

**Table 3:** **The ERI model in the surveys**

Dimension of ERI model	Dimension in overview	NL	DK	NO	FI	ES	DE	EU
**Effort**	Quantitative demands - work pace	**√**	**√**	**√**	**√**	**√**	**√**	**√**
	Quantitative demands - amount	**√**	**√**	**√**	√*	**√**	**√**	**√**
	Interruptions	-	-	**√**	**√**	-	**√**	**√**
	Cognitive demands	**√**	**√**	-	-	**√**	**√**	**√**
**Reward - money & promotion**	Career development	**√**	**√**	**√**	**√**	-	-	**√**
	Financial rewards	**√**	-	**√**	**√**	-	**√**	**√**
**Reward - esteem**	Contact with supervisor/social support by supervisor	**√**	**√**	**√**	**√**	**√**	**√**	**√**
	Contact with co-workers/social support by co-workers	**√**	**√**	**√**	**√**	**√**	**√**	**√**
	Justice/discrimination	**√**	**√**	**√**	**√**	**√**	-	**√**
	Recognition	-	**√**	**√**	-	-	-	-
**Reward - status control & job security**	Job security	**√**	**√**	**√**	**√**	**√**	**√**	**√**

*Notes*: √ = dimension is available in the survey; - = dimension is not available in the survey; * In Finland, the dimension "quantitative demands – amount" is assessed by asking participants about the number of compensated and non-compensated overtime at work.

NL = Netherlands; DK = Denmark; NO = Norway; FI = Finland; ES = Spain; DE = Germany; EU = Europe.

Dimensions of the DCS-model are to a great extent covered in the surveys; it is solely one dimension - "role conflict" - that is assessed in three surveys only. Whereas quantitative demands – amount" are assessed with items asking about work piling up, having too much to do and working to tight deadlines in six surveys, the dimension is assessed by asking participants about the number of compensated and non-compensated overtime at work in Finland.

For the ERI model, the picture is more diverse: Whereas the effort dimension is only fully covered in Germany and Europe, the three aspects of the reward dimension are fully covered only in Norway. Among the three aspects of rewards, the Spanish survey doesn’t cover the aspect of "money and promotion" at all whereas in the other countries, this aspect is at least partly covered. The aspect "job security" is fully and the aspect "esteem" at least partly covered in all seven surveys.

At the same time, our analyses reveal that a variety of working conditions outside the two prominent models on work and stress are actually monitored in large national surveys: more than half of the dimensions in our taxonomy stem from other sources than DCS or ERI. A majority of these dimensions, especially those present in all or nearly all surveys e. g. "emotional demands" (in all seven surveys) and "meaning of work" (in six out of seven surveys), is covered by the COPSOQ as can be seen in Table 1. Of the five dimensions that are neither covered by DCS, ERI nor COPSOQ, only one is covered in six out of seven surveys: "restructuring" The other four dimensions are only covered in some surveys, e.g. "workplace innovation" (three out of seven surveys) and "organizational influence" (four out of seven surveys).

## Discussion

This paper aimed at answering two questions regarding monitoring of working conditions in Europe. First, which dimensions of the psychosocial working environment are monitored in six national and one European wide survey and are comparisons between survey results possible. Second, to which extent do these dimensions overlap with the most prominent models on work and health – the DCS and the ERI – and to which extent are dimensions outside these models with possible relations to health outcomes covered.

With regard to the first question, we can state that there is a large overlap of dimensions covered in the seven surveys. A number of dimensions were measured in most surveys: quantitative demands (amount and work pace), emotional demands, degrees of freedom, influence/decision authority, possibilities for development/skill discretion, variation/repetition, meaning of work, social support (co-worker, supervisor), social community at work, restructuring, job security, work-life balance, justice/discrimination and offensive behaviour/violence & harassment. However, the items used to assess these concepts differ between the surveys – sometimes rather slightly, and sometimes more profoundly. Furthermore, different items tap into very different aspects of the same concept so coverage of the dimensions differs a lot, e.g. the dimension "possibilities for development/skill discretion" can be measured by use of available skills, developing available skills, learning new skills, being creative or attending trainings. For comparative purposes, e.g. with regard to prevalence, this poses a problem: It is impossible to say where differences between two countries that use differently formulated items stem from.

With regard to the second question, we can state that both the DCS-model and – to a slightly lesser extent – the ERI–model are covered in most countries. At the same time, more than half of the dimensions present in the surveillance systems are not part of these two prominent models on work and health. The "non-model-dimensions" being measured in most surveys were: emotional demands, degrees of freedom, meaning of work/commitment to the workplace, social community at work, restructuring, work-life balance and offensive behaviour/violence & harassment.

We found that dimensions assessed in the surveys are to a large extent the same, indicating that similar themes are covered within the field of psychosocial working conditions across Europe. Consequently, one can assume that interests in the countries are to a considerable extent similar and that the same themes are of relevance for the social partners.

At the same time, we see a lot of dissimilarities in the specific formulation of items used to assess these dimensions. One example is the dimension ‘quantitative demands - amount’: Danish workers are asked if they have an unevenly distributed work burden and if they can accomplish their work tasks. In Finland, the dimension is assessed by asking workers about the number of compensated and non-compensated overtime at work. It is also interesting to see that in some surveys, the items require a description of the working conditions whereas in other surveys, workers are encouraged to report their evaluation of the impact of their working conditions on them. E.g. for the dimension "financial rewards", workers in Norway are asked whether their salary is appropriate for their effort and performance at work, whereas workers in Germany, Finland and the Netherlands are asked if they are satisfied with their salary. Differences in formulation are presumably a result of a strong national focus in the development of the surveys with rather little coordination between the countries.

We have not investigated the theoretically derivable factorial structure of the dimensions underlying the items used in the seven surveys. Therefore, we cannot know for certain that the items of e.g. the DCS represent three distinct factors. This could be considered a limitation of our study. However, we did not carry out such an analysis because of the large differences in the formulation of items in the surveys included in the present paper (see previous paragraph).

Comparisons between countries could be facilitated by aligning the formulation of items – however, changes in the formulation of items would make it impossible to analyse trends per country as such an analysis requires the usage of the same item over time. Apart from that, because in each country different partners have a say in the design of the surveys, changes cannot be easily implemented. In the end, one has to find a balance between comparability between and within countries.

It is surprising to see how much the two prominent models on work and health are covered in the surveys when bearing in mind that the primary aim of monitoring is not to measure scientific concepts. From our analyses we can conclude that the DCS-model is indeed very well covered in the surveys: For the dimension "demand", two out of three aspects (quantitative demands - work pace & amount) are available in all countries; for the dimension "control", two out of three aspects – the core aspects: possibilities for development/skill discretion and influence/decision authority – are available in all countries; and for the dimension "support", both aspects (supervisor and co-worker) are available in all countries.

Coverage is not as large but still satisfactory for the ERI-model: The dimension "effort" is at least partly covered in all countries as is one of the three aspects of "reward – esteem". Another aspect, "reward – job security", is covered across all countries. The third aspect "reward – money and promotion" is only fully covered in four surveys and not covered at all in one country. Nevertheless, conclusions about relations of ERI dimensions and health outcomes can be drawn from the surveys.

In the scientific community, especially the DCS-model and the ERI-model have dominated research on "work stress". Consequently, a large number of studies are available on DCS’ and ERI’s relations to health outcomes. Fewer studies are available on the psychosocial dimensions outside these two models; an example is the research on risk factors for ischemic heart disease and mental health [9, 12, 13]. Our analyses reveal that a variety of working conditions outside these two models are actually monitored in large national surveys, constituting more than half of the dimensions covered altogether. Some of these dimensions are present in nearly all surveys. They present therefore an opportunity for new research if linked to health outcomes.

At the same time, not all aspects inherent in the two prominent models are covered in all surveys, as is for example the case for "role conflict" (see Table 2), an aspect of demands in the DCS assessed with one item [33]. Role conflict has in recent papers been reported to be prospectively associated with health outcomes [34, 35], indicating that this dimension is important to measure in the surveys.

Norway covers all core concepts included (see Tables 2 and 3) except "cognitive demands". This is supposedly based on the fact that the questionnaire was revised in 2009, now including new dimensions based on the review on surveillance systems provided by Dollard, Skinner, Tuckey and Bailey [5]. Hence, a systematic approach to survey revision seems to lead to a wider coverage of dimensions – established ones as well as emerging ones. The overview in the present paper might give input to planning of new and revisions of existing surveys as well.

### Methodological challenges

For some items, it proved to be challenging to sort them into one specific category. These were items that refer to two or more dimensions of the taxonomy, tapping different aspects at the same time and usually very specific for one country. The latter can be regarded as an indication of these items being genuine in trying to capture emerging risks that have only recently gained interest of the social partners or the scientific community and have not become established concepts yet. One such example is the Norwegian item "Do you sometimes have to perform tasks that you do not feel adequately trained to do?". This item could be regarded as assessing a very specific demand – a "learning demand" – not assessed in any other country. We did not find it feasible to include a large number of very specific dimensions just available for one country into the taxonomy as this posed a risk to a parsimonious structure allowing comparisons between surveys. In difficult cases like this, we agreed upon one dimension. The aforementioned item was placed under the dimension "role conflict" after consideration of possible other dimensions, e.g. "predictability". Hence, our procedure could potentially have led us to establishing too few dimensions in our overview.

Apart from the EWCS study, we have mainly analysed surveys from the northern and western European countries based on our restriction to studies carried out in the member countries of the PEROSH group "Survey development and cross culture methodology" with Spain as the only country from southern Europe. It is possible that in other European countries - as well as in other countries outside Europe - further working conditions have a stronger impact on the workers’ health as for example assessed in a scale on precarious work developed by Vives, Amable, Ferrer, Moncada, Llorens, Muntaner, Benavides & Benach [36], depending e.g. on the welfare systems in the countries. First results supporting this point were presented by Dragano, Siegrist & Wahrendorf [37]. Prospective scientific studies in a large number of countries are needed in order to evaluate which dimensions are important for health.

## Conclusions

We believe we with the present study have given a needed overview over OSH-monitoring in Europe. We hope the overview will provide input to the revisions and updates of individual countries’ surveys. Our study also found that the dimensions of DCS and ERI are covered in most surveys plus a large variety of psychosocial dimensions not covered in these models. We believe the latter provide encouraging possibilities for research on possible health effects of psychosocial working conditions not being part of the DCS and ERI models but of interest to political stakeholders as for example "emotional demands" and "restructuring". The surveys we have compared and the data that is collected by means of these questionnaires offer a basis for analysing the potential impact of these working conditions on the workers’ health in large samples with a prospective design. One option would be to investigate the predictive validity of dimensions not covered by the classical models over a long period of time in those surveys with a longitudinal design where the development in health or labour market participation can be followed in individuals, either through questionnaires [38, 39] or in registers [40]. Another option would be the development of job exposure matrices (JEM) based on the national data on working conditions. Linking the combined exposure information with longitudinal health information via occupational codes would allow for an estimation of the dimensions’ long-term impact on the workers’ health, based on the condition that there is sufficient variation in working conditions between the occupations. The monitoring and the scientific community could benefit from one another by combining their efforts in the research of possible health effects of these dimensions, yielding a more comprehensive picture of the psychosocial work environment.

## Electronic supplementary material

Additional file 1: **Overview of all dimensions and items assessing psychosocial working conditions in the seven surveys.** (PDF 69 KB)

## Abbreviations

DCS: Demand-control-support-model
ERI: Effort-reward-imbalance-model
OSH: Occupational safety and health
BAuA: Federal Institute for Occupational Safety and Health (Germany)
BIBB: Federal Institute for Vocational Education and Training (Germany)
PEROSH: Partnership for European Research in Occupational Safety and Health
ENCT: Spanish National Working Conditions survey
NEA: National Working Condition Survey in the Netherlands
FNWHS: Finnish National work and health survey
LKU: Norway - Survey of living conditions - Working environment
DWECS: Danish Work Environment Cohort Study
BIBB/BAuA: German Labour Force Survey
EWCS: European Working Conditions Survey
COPSOQ: Copenhagen Psychosocial Questionnaire
QPS Nordic: General Nordic Questionnaire for Psychological and Social Factors at Work
NL: Netherlands
DK: Denmark
NO: Norway
FI: Finland
ES: Spain
DE: Germany
EU: Europe
JEM: Job exposure matrix.
