# Supplementary material for: Dimensional comparability of psychosocial working conditions as covered in European monitoring questionnaires
Source: BMC Public Health. 2014 Dec 9;14:1251. doi: 10.1186/1471-2458-14-1251 (PMC4295265; doi:10.1186/1471-2458-14-1251)
Supplement: Supplementary file 1 — Additional file 1: Overview of all dimensions and items assessing psychosocial working conditions in the seven surveys. (PDF 69 KB) [file 12889_2014_7371_MOESM1_ESM.pdf]

	Country	Netherlands	Denmark	Norway	Finland	Germany	Spain	Europe
**Domain**	**Dimension**	**Netherlands Working Conditions Survey (NEA)**	**Danish Work Environment Cohort Study (DWECS)**	**Survey of living conditions: Working environment (LKU)**	**The Finnish National Work and Health Survey (FNWHS)**	**German Labour Force Survey (BIBB/BAuA)**	**Spanish National Working Conditions survey (ENCT)**	**European working conditions survey (EWCS)**
**demands at work**	**demands - general (work load)**	Do you need to work extra hard?			Is your work mentally light, fairly light, a bit strenuous, quite strenuous or very strenuous ?	Do you generally feel you can cope with the requirements due to the work volume or the workload or do you tend to be overtaxed or underchallenged?	To what extent does your work imply…having a lot of work and feeling overwhelmed?	
		Is your work hectic? [no, sometimes, often, always]				Do you feel you can generally cope with the requirements placed on your professional knowledge and skills in your occupation as a < insert occupation from F100-102 > or do you tend to be overtaxed or underchallenged?		
	**quantitative demands - work pace**	Do you have to work at a high pace?	Is it necessary to work very quickly?	How often is it necessary to work at a rapid pace?	How often do you have to rush to get work done?	How frequently does it occur in your work that you have to work very fast? Is that a load situation for you?	To what extent does your work imply…working very fast?	And, does your job involve working at very high speed?
		Do you need to work very fast?					Mark how much the following aspects of your work bother or annoy you: the work rhythm	
	**quantitative demands - amount**	Do you need to do a lot of work?	Is your work unevenly distributed so that it can pile up?	How often do you have too much to do?		How often does it occur that you have to work under strong pressure from deadlines or performance? Is that a load situation for you?	Mark how much the following aspects of your work bother or annoy you: the work load	And, does your job involve working to tight deadlines?
		Do you work under great time pressure?	How often does it occur that you do not accomplish all your work tasks?	Do you sometimes have so much to do that you have to miss lunch, work longer than your normal working hours or take work home with you?			To what extent does your work imply…working with very strict or short deadlines?	You have enough time to get the job done.

Country	Netherlands	Denmark	Norway	Finland	Germany	Spain	Europe
			How well does the following description fit your current job? … There is normally not time to perform tasks properly.				
**emotional demands**	Does your work get you into emotionally difficult situations?	Is your work emotionally draining?	In your work, to what extent do you need to deal with strong feelings such as sorrow, anger, desperation, frustration and so on from customers, clients or other people who are not employed at your workplace?	How often do you find yourself in situations at work that arouse negative feelings in you such as anger, hate, fear or shame?	How often does it occur that your activity produces situations which affect you emotionally?	Mark how much the following aspects of your work bother or annoy you: the relationships with my workmates	You get emotionally involved in your work.
	Is your work emotionally demanding?	Are you affected emotionally by your work?				Mark how much the following aspects of your work bother or annoy you: the relationships with my bosses	
	Do you get emotionally involved with your work?	Does your work put you in emotionally draining situations?				Mark how much the following aspects of your work bother or annoy you: the relations with people not employed by the company: costumers, passengers, students, patients, etc.	
**demands on hiding emotions**			In your work, to what extent do you need to conceal negative feelings such as anger, irritation, frustration and so on for customers, clients or other people who are not employed at your workplace?				Your job requires that you hide your feelings.
**cognitive demands**	Does your work require you to think very hard?	To what degree do you have to survey a large amount of information in order to carry out your work?			How frequently does it occur in your work that you have to keep an eye on different tasks or processes at the same time? Is that a load situation for you?	To what extent does your work imply…having to carry out complex, complicated or difficult tasks?	Generally, does your main paid job involve complex tasks?

Country	Netherlands	Denmark	Norway	Finland	Germany	Spain	Europe
	Does your work require that you keep your mind on your job?				How frequently does it occur in your work that even a small mistake or slight inattention can result in major financial losses? Is that a load situation for you?	Mark how much the following aspects of your work bother or annoy you: the difficulty or complexity of the tasks	If you make mistakes in your work, could it cause financial loss to your company?
						I consider my work to be intellectually demanding.	Generally, does your main paid job involve ... meeting precise quality standards?
						To what extent does your work imply…having to carry out several tasks simultaneously?	Generally, does your main paid job involve… assessing yourself the quality of your own work?
**sensorial demands**	Does your work require a lot of your attention?					To what extent does your work imply…maintaining a high or very high attention level?	
**contact with clients, suppliers etc.**	How often do you have contact with customers/clients/patients/pupils/passengers etc.?	In connection with your work, do you have anything to do with people who are not employed at your work place (for example, customers, clients, passengers, students)?	In your job, what proportion of your time do you spend face to face or speaking on the phone to clients or customers?			To what extent does your work imply…working directly with people who are not employed by your company: customers, passengers, students, patients, etc.?	Please tell me, using the same scale, does your main paid job involve dealing directly with people who are not employees at your workplace such as customers, passengers, pupils, patients, etc.?
	How often do you have contact with suppliers/sub-contractors?						Please tell me, using the same scale, does your main paid job involve … handling angry clients?
**interruptions**			How often do you experience interruptions that disturb your work?	Do you have to interrupt work you are doing to carry out something else that has cropped up or to do something that is more urgent?	How frequently does it occur in your work that you are disturbed or interrupted in your work, e.g. by colleagues, poor material, machine faults or telephone calls? Is that a load situation for you?		How often do you have to interrupt a task you are doing in order to take on an unforeseen task?

	Country	Netherlands	Denmark	Norway	Finland	Germany	Spain	Europe
								For your work, are these interruptions [disruptive, without consequences, positive]?
								Generally, does your main paid job involve solving unforeseen problems on your own?
**work organization & job content**	**degrees of freedom**	Are you able to take time off work when you want to?	In your work place is there opportunity to take care of your family when it is necessary?	Are you free to choose when to take a break from your work: e.g. to stretch your legs or take a breather in some other way?	How often do you have to be flexible in your working times either because the job requires it or your supervisor asks you to?	Do you manage to allow for your family and private interests in planning your working time?	Can you chose or modify...the distribution and/or duration of the breaks?	Would you say that for you arranging to take an hour or two off during working hours to take care of personal or family matters is ... ? [not difficult at all, not too difficult, somewhat difficult, very difficult]
					Can you affect the length of your working day ?	How frequently does it occur that you can decide when to take break?		On the whole, is your pace of work dependent, or not, on the work done by colleagues?
								On the whole, is your pace of work dependent, or not, on direct demands from people such as customers, passengers, pupils, patients, etc.?
								On the whole, is your pace of work dependent, or not, on numerical production targets or performance targets?
								On the whole, is your pace of work dependent, or not, on automatic speed of a machine or movement of a product?
								On the whole, is your pace of work dependent, or not, on the direct control of your boss?

Country	Netherlands	Denmark	Norway	Finland	Germany	Spain	Europe
							You can take a break when you wish?
**influence / decision authority**	Are you able to decide for yourself how to do your work?	Do you have a significant influence in the decision making at your work?	To what extent are you free to decide your own tasks?	Can you influence things that affect you at your workplace ?	How frequently does it occur that you can plan and organise your own work yourself?	To what extent can you chose or modify…tasks order?	I make the most important decisions on how to run my business.
	Are you able to decide for yourself in which order to do your work?	Are you able to perform your work at a quality level that you are completely satisfied with?	To what extent are you free to decide how to go about doing your work?	What about the amount of work - can you influence it at your place of work?	How frequently does it occur ...that you have an influence on the work volume allocated to you?	To what extent can you chose or modify…working method?	Are you able to choose or change your order of tasks?
	Are you able to influence the pace in which you work?	Do you participate in the planning of your work (for example, what needs to be done, how it will be accomplished, or who you will work with)?	To what extent can you influence decisions that are important to your work?		How frequently does it occur in your work that an exact number, a certain minimum output or the time is specified to you in order to perform a certain task?	To what extent can you chose or modify…work pace?	Are you able to choose or change your methods of work?
		Do you have any influence on the amount of work you do?	To what extent can you set your own work pace?		How frequently does it occur in your work that the performance of the work is specified to you down to the last detail? Is that a load situation for you?	To what extent does your work imply…having the oportunity to do what you can do best at work?	Are you able to choose or change your speed or rate of work?
		Do you have any influence in WHAT you do at work?					You are consulted before targets for your work are set.
		Do you have any influence in WHO you work with?					You have a say in the choice of your working partners.
							You can influence decisions that are important for your work.
							Who decides the division of the tasks? [Your boss or manager / They are decided by people who are rotating tasks]

Country	Netherlands	Denmark	Norway	Finland	Germany	Spain	Europe
**possibilities for development / skill discretion**	Does your job require to learn new skills?	Do you have the opportunity to learn something new through your job?	In your job, how good are your opportunities to … develop your skills in the areas that interest you?	What kind of opportunities do you have to develop yourself professionally in your present workplace ?	How frequently does it occur in your work that things are demanded from you which you have not learned or which you do not master? Is that a load situation for you?	To what extent can you...learn new things?	Generally, does your main paid job involve learning new things?
	Does your job require creativity?	Can you use your abilities or skills in your work?	In your job, how good are your opportunities to make use of the skills, knowledge and experience that you have gained through your education and past work?	Generally speaking is training arranged at your place of work: training is not needed at my workplace, very well, fairly well, fairly poorly or extremely poorly ?	How frequently does it occur in your work that you are faced with new tasks which you first have to try and understand and become familiar with? Is that a load situation for you?		Which of the following alternatives would best describe your skills in your own work? [I need further training to cope well with my duties / My present skills correspond well with my duties / I have the skills to cope with more demanding duties]
	Do you need to come up with solutions yourself?	Does your work require that you are full of initiative?	How often does your job require you to acquire new knowledge and skills?				
**variation / repetition**	Doed your work vary?	Is your work varied?	Does your job consist of constantly repeated tasks, meaning that you do the same thing hour after hour?		To what extent does your work imply…carrying out very repetitive and very short tasks?	To what extent does your work imply…carrying out monotonous tasks?	Generally, does your main paid job involve monotonous tasks?
		Does your work require that you repeat the same tasks several times an hour?					Please tell me, does your job involve short repetitive tasks of less than ... 1 minute?
							Please tell me, does your job involve short repetitive tasks of less than… 10 minutes?
							Does your job involve rotating tasks between yourself and colleagues?
							Do the [rotating] tasks require different skills?

Country	Netherlands	Denmark	Norway	Finland	Germany	Spain	Europe
**meaning of work / commitment to the workplace**		Are your work tasks meaningful?	How often do you feel motivated and enthusiastic about your work?	How often are you enthusiastic about your work?	How often do you have the feeling that your work is important?	To what extent does your work imply…having the feeling of doing something useful?	You have the feeling of doing useful work.
		Do you feel motivated and involved in your work?		How often do you feel yourself to be strong and dynamic at work?			
		Do you feel that the work you do is important?					
**organizational influence / organizational decision latitude**			At your workplace, if you state your opinions about working conditions, do you feel that you get listened to?	Can you talk about joint working, objectives and their achievement at your workplace ?		To what extent does your work imply...applying your own ideas to your work?	You are involved in improving the work organisation or work processes of your department or organisation.
				At my place of work decisions are made on the basis of correct information.			At your workplace, does management hold meetings in which you can express your views about what is happening in the organisation?
				Decisions made at my place of work are consistent i.e. the rules are the same for everyone.			
				Is the atmosphere at your place of work more supportive and encouraging of new ideas or more prejudiced and holding fast to old ways of doing things ?			
**workplace innovation**				Is time used to develop new ideas at your workplace?	How frequently does it occur in your work that you improve previous processes or try out something new?		You are able to apply your own ideas in your work.
							You are involved in improving the work organisation or work processes of your department or organisation.

	Country	Netherlands	Denmark	Norway	Finland	Germany	Spain	Europe
**interpersonal relations & leadership**	**contact with supervisor / social support by manager**	My supervisor pays attention to what I say.	How often are your immediate superiors willing to listen to your work-related problems?	If you need it, how often can you get the support and help of your immediate superior with your work?	Do you get help and support from your line manager ?	And how often do you receive help and support for your work from your direct supervisor when you need it?	To what extent can you...obtain help from your superiors / bosses if you ask for it?	Your manager helps and supports you.
			How often do you receive help and support from your immediate superiors?	Do you get feedback from your superiors on how you have performed your work?				In general, your immediate manager / supervisor provides you with feedback on your work.
	**contact with co-workers / social support by co-workers**	How often do you have contact with colleagues from you own team?	How often do you receive help and support from your colleagues?	If you need it, how often can you get the support and help of your colleagues with your work?	Do you receive support and help from your colleagues when you need it?	How often do you feel that the co-operation between you and your work colleagues is good?	To what extent can you...obtain help from your workmates if you ask for it?	Your colleagues help and support you.
		How often do you have contact with colleagues from another team?	How often are your colleagues willing to listen to your problems with work?		Are your relations with your colleagues at your workplace generally very good, moderately good, not good, but not bad either, a little difficult or bad ?	How often do you receive help and support for your work from colleagues when you need it?		
		My colleagues show a personal interest in me.						
		My colleagues are friendly.						
		My colleagues are good at their job.						
	**teamwork / social community at work**	My colleagues help to get the job done.		To what extent do you feel a sense of belonging to the company or organisation at which you work?	Do you work in a permanent working group with a joint role and which has the opportunity to plan its work itself and decide on the division of wok within the group ?	How often does it occur that you feel part of a community at your workplace?	To what extent do you agree with the following statement: the personal relations are positive and induce cooperation?	Do you work in a group or team that has common tasks and can plan its work?
					Is the atmosphere at your place of work more strained and tense or more relaxed and pleasant ?			For the team in which you work mostly, do the members decide by themselves on the division of tasks?

Country	Netherlands	Denmark	Norway	Finland	Germany	Spain	Europe
							For the team in which you work mostly, do the members decide by themselves who will be head of the team?
							For the team in which you work mostly, do the members decide by themselves the timetable of the work?
**quality of leadership**	My supervisor looks out for the well-being of the employees.	To what degree would you say that your immediate superior at your work ensures that the individual employee has good development opportunities?					In general, your immediate manager / supervisor respects you as a person.
	My supervisor helps to get the job done.	To what degree would you say that your immediate superior at your work highly prioritizes job satisfaction?					In general, your immediate manager / supervisor is good at resolving conflicts.
	My supervisor is able to let people work with each other.	To what degree would you say that your immediate superior at your work is good at planning the work?					In general, your immediate manager / supervisor is good at planning and organising the work.
		To what degree would you say that your immediate superior at your work is good at solving conflicts?					In general, your immediate manager / supervisor encourages you to participate in important decisions.
**predictability**		In your workplace, are you informed about important decisions, changes, and future plans with plenty of notice?	Does your employer give you plenty of notice of important decisions, changes and future plans?		How often does it occur that you are not informed in good time about far-reaching decisions, changes or plans for the future?		
		Do you receive all the information you need in order for you to do your job well?			How often does it occur that you do not receive all the necessary information to enable you to perform your work properly?		

Country	Netherlands	Denmark	Norway	Finland	Germany	Spain	Europe
**recognition**		Is your work recognized and valued by the management?	To what extent do you agree or disagree with the following statement: In relation to my efforts and performance, I get the respect and recognition that I deserve at work.				
		Is your work recognized and valued by society?	Are your work achievements appreciated by your immediate superior?				
**career development**	Was your position extended in the last two years?	Are there good prospects in your job?	Do you have opportunities for promotion or career development at your company?	What kind of opportunities do you have to develop yourself professionally in your present workplace?			Do you agree or disagree with the following statements describing some aspects of the training?... The training has helped me improve the way I work.
	Did you make an advancement within your company in the last two years?						… I feel that my job is more secure because of my training.
	In the last two years, did your company pay for an exernal education or training?						… I feel my prospects for future employment are better.
							My job offers good prospects for career advancement.
**role conflict**		How correct or incorrect are the following statements about your role in your work? Conflicting demands are placed on me in my work.	How often do you receive incompatible requests from two or more people?				Your job involves tasks that are in conflict with your personal values.
			How often are you given assignments without adequate resources to complete them?				
			How often do you have to do things that you feel should be done differently?				

Country	Netherlands	Denmark	Norway	Finland	Germany	Spain	Europe
			Do you sometimes have to perform tasks that you do not feel adequately trained to do?				
**role clarity**		How correct or incorrect are the following statements about your role in your work? I clearly know what my areas of responsibility are.	How often is it unclear what is expected of you in your job?	Does your work have clear objectives ?			You know what is expected of you at work.
**financial rewards**	How satisfied are you regarding these aspects of your present job: … good salary?		To what extent do you agree or disagree with the following statement? My salary is appropriate to my efforts and performance at work.	Is your present salary satisfactory, unsatisfactory or neither?	How satisfied are you...with the income from this occupation <insert occupation from F100-102 >?		I am well paid for the work I do.
				With regard to the evolution of your wages/salary are you satisfied, unsatisfactory or neither?			
**safety culture**	At my work they listen to suggestions of employees about ways to improve the safety.	Do you agree with the following statement about your work place: The management expects that the employees take risks when the work schedule is pressured.				To what extent do you consider yourself to be correctly informed regarding the health and safety risks at your work place?	Regarding the health and safety risks related to performance of your job, how well informed would you say you are?
	At my work employees get sufficient information occupational safety.	The management is not concerned about whether the work is carried out safely as long as it is completed on time.				Have you received any training or information regarding the health and safety risks at your work place during the last two years?	
	At my work saftey issues are dealt with swiftly.	Have you been informed about how your job can be carried out safely?					
	At my work occupational safety is encouraged.						

Country	Netherlands	Denmark	Norway	Finland	Germany	Spain	Europe
	At my work they abide by the rules about working safely.						
restructuring	In the last year, were there any changes in your workplace (take-over/reorganisation/downsizing/merger/etc.)? [A major restructuring? A takeover by another organisation? A takeover of another organisation? A downsizing without the loss of jobs? A downsizing with the loss of jobs? A merger with another company? Outsourcing of supportive services? Relocation of activities to another country? Automatization of activities? None of the above?]	In the last year, has your workplace been taken over by another employer?	Has the company where you currently work implemented downsizing at any point during the past three years?	During the past 3 years have there been changes at your place of work, for example in production, working methods or working arrangements which have changed your job, your responsibilities or workload?	Now please tell me whether the following changes were made in the last two years in your <direct working environment>. Were in the last two years…new production or process technologies introduced in this time in your direct working environment?		During the last 3 years have the following changes occurred at your current workplace which affected your immediate working environment: … new processes or technologies were introduced?
			Has the company carried out any restructurings over the past three years that have affected your work situation, but that have not involved staff cuts? [Yes, in my department; Yes, in other departments at the company; no]	What have these changes been or what have they been concerned with?	Were in the last two years…new computer programs introduced? This does not mean new versions of existing programs.		During the last 3 years have the following changes occurred at your current workplace which affected your immediate working environment:… substantial restructuring or reorganisation was carried out?
			What impact have the downsizing had? Would you say that the impact was very big, big, small or very small or non-existent on your everyday work? If several rounds of staff cuts have been implemented, choose the one that had the biggest impact on you.		Were in the last two years…new machines or equipment introduced in your direct working environment?		If you compare your current situation with that of January 2009, have you experienced a change in the following aspects of your work: … the number of hours you work per week?

Country	Netherlands	Denmark	Norway	Finland	Germany	Spain	Europe
			Has the company carried out any restructurings over the past three years that have affected your work situation, but that have not involved staff cuts?		Were in the last two years… new or considerably changed products or materials introduced in your direct working environment?		If you compare your current situation with that of January 2009, have you experienced a change in the following aspects of your work:… your salary or income?
			(if yes) What impact did the restructuring have? Would you say that the impact was [very big, big, small or very small or non-existent on your everyday work?] If several rounds of restructuring have been implemented, choose the one that had the biggest impact.		Were in the last two years… new or considerably changed services provided?		
			In relation to the most recent downsizing, I would like you to say to what extent you agree with the following statement…. I had the opportunity to talk to my immediate superior about the impact of the changes on me.		Were in the last two years… major restructuring or reorganisation measures undertaken which affected your direct working environment?		
			I have received the necessary training to perform my new tasks and roles? (the question is also asked in relation to restructuring)		Were in the last two years… jobs lost or dismissals made <in the last two years / in this time> in your direct working environment?		
			My wishes and suggestions were taken into account in the planning and implementation of the changes. (the question is also asked in relation to restructuring)		Were in the last two years… increasingly more freelance workers, assistants, interns or temporary workers deployed?		

	Country	Netherlands	Denmark	Norway	Finland	Germany	Spain	Europe
				In the past three years, have staff cuts at your company caused you to resign, be dismissed or leave your job after receiving a severance package? [Yes, resigned from a job; yes, was dismissed; yes, left after receiving a severance package; no]		Have you had a new direct supervisor <in the last two years / in this time >?		
				Prior to leaving, were you given the opportunity to talk to your immediate superior (employer) about what impact the staff cuts would have on you?		Has the variety of tasks increased, remained the same or decreased in this time?		
				Have you been transferred to another job at your company/enterprise against your will?		Have the professional requirements of your work increased, remained the same or decreased in this time?		
**work-individual interface**	**job security**	Are you a risk of losing your job?	Do you worry that the following may happen to you: You become unemployed?	Do you believe that you are at risk of losing your job due to your company closing, downsizing or other reasons over the coming 3 years? [Yes, due to it closing; Yes, due to downsizing; Yes, for other reasons; no]	Do you think the the financial situation at your workplace at the moment is completely stable and secure, to some extent stable and secure, a little uncertain or very uncertain?	How do you assess the economic situation of the company where you work?	Mark how much the following aspects of your work bother or annoy you: the risk to lose the job.	I might lose my job in the next 6 months.
		Do you worry about losing your job?	Do you worry that the following may happen to you: You are transferred to another job against your will?	Do you believe that you are at risk of being transferred to another job at your company / enterprise against your will?	How probable do think it is that you will become unemployed or laid-off within the year, is it unlikely, possible, fairly certain, or certain?	How high do you assess the risk that in the near future your contract work for the company for which you work the most will be ended? [very high; high; low; or is there no risk of that at all?]		If I were to lose or quit my current job, it would be easy for me to find a job of similar salary.

	Country	Netherlands	Denmark	Norway	Finland	Germany	Spain	Europe
			Do you worry that the following may happen to you: You become unnecessary because of the implementation of "new technology"?	Do you think that there will be a need for employees with your skills and experience in the labour market in five years' time?		[for self-employed]. How high do you assess the risk that you will have to shut down your company in the near future?		
			Do you worry that the following may happen to you: You have difficulty finding a new job with your qualifications?					
	**work-life balance**	Do you miss or neglect your family activities because of your work?	Overall, how do you think your work affects your private life?	How often do the requirements of your job disturb your home and family life?	People sometimes find it hard to reconcile the conflicting demands of home and work. Do you have difficulties in concentrating on work because of things at home ?	Do you manage to allow for your family and private interests in planning your working time?	In general, does your work schedule allow you to combine work and family, social commitments?	In general, do your working hours fit in with your family or social commitments outside work very well, well, not very well or not at all well?
		Do you miss or neglect your work because of family activities?	In general, how do you feel your private life affects your work?	How often are you called or contacted by someone from work outside working hours, in order to ask you about something related to your work?	How often do you feel that you are neglecting things at home because of your job?			
					How has your personal life been outside work during the past six months? Has it been easy, quite easy, quite hard, hard or extremely hard?			
**values at the workplace**	**trust**			How well does the following description fit your immediate superior: Your immediate superior treats employees with trust and respect?				
				Do you risk being met with reluctance from your superiors if you make critical comments about the working conditions at your workplace?				

Country	Netherlands	Denmark	Norway	Finland	Germany	Spain	Europe
			Do you risk being met with reluctance from your colleagues if you make critical comments about the working conditions at your workplace?				
**justice / discrimination**	At your work is there any discrimination on the basis of gender?	In your work place is there room for employees with different disabilities and handicaps?	Does your immediate superior treat employees fairly and equally?	Does your immediate line manager deal with employees fairly and equally?		Nationality discrimination	Over the past 12 months, have you been subjected at work to … age discrimination?
	At your work is there any discrimination on the basis of race?			Decisions made at my place of work are consistent i.e. the rules are the same for everyone.		Age discrimination	Over the past 12 months, have you been subjected at work to… discrimination linked to race, ethnic background or colour?
	At your work is there any discrimination on the basis of religious beliefs?			In comparison to other employees, are immigrants at your place of work treated completely equally, fairly equally, fairly unequally, or extremely unequally?		Sexual or Gender discrimination	Over the past 12 months, have you been subjected at work to… discrimination linked to nationality?
	At your work is there any discrimination on the basis of sexual preference?			Are men and women at your workplace treated completely equally, fairly equally, fairly unequally, or extremely unequally?		Racial or ethnic discrimination	Over the past 12 months, have you been subjected at work to… discrimination on the basis of your sex?
	At your work is there any discrimination on the basis of age?			Is the inequality directed towards men or women?		Religious discrimination	Over the past 12 months, have you been subjected at work to… discrimination linked to religion?
				Are different aged people at your workplace treated completely equally, fairly equally, fairly unequally, or extremely unequally?		Disability discrimination	Over the past 12 months, have you been subjected at work to… discrimination linked to disability?

	Country	Netherlands	Denmark	Norway	Finland	Germany	Spain	Europe
					Is the inequality directed towards younger people or older people ?		Sexual orientation discrimination	Over the past 12 months, have you been subjected at work to… discrimination linked to sexual orientation?
					Does your immediate line manager deal with employees fairly and equally?			
**offensive behaviour**	**offensive behaviour / violence & harassment**	Unwanted sexual attention from clients (or patients, students, passengers etc.)	In the last 12 months, have you been subjected to harassment in your work place? (That is to say that over several months you have been subjected to uncomfortable or degrading treatment which is difficult to defend yourself against)	Do you yourself sometimes get bothered or teased in an unpleasant way by your colleagues?	Mental violence means continual repeated teasing, bullying or offensive behaviour. Have you earlier or are you at the moment been the object of mental violence and teasing in your own place of work?		Have you been subject to any of the following during the last twelve months? .. Physical violence threats / Physical violence from other people at your workplace	Over the last month, during the course of your work have you been subjected to … verbal abuse?
		Unwanted sexual attention from superiors or colleagues.	In the last 12 months, have you been subjected to unwanted sexual attention in your work place?	Do you yourself sometimes get bothered or teased in an unpleasant way by superiors?	Where does the teasing mainly come from?		Have you been subject to any of the following during the last twelve months?.. Physical violence from other people at your workplace	Over the last month, during the course of your work have you been subjected to… unwanted sexual attention?
		Intimidation by clients (or patients, students, passengers etc.)	In the last 12 months, have you been subjected to violent threats in your work place?	Do you sometimes receive unwanted sexual attention, comments, etc. at your workplace?	Have you experienced sexual harassment at your place of work during the past 12 months?		Have you been subject to any of the following during the last twelve months?.. Sexual harassment	Over the last month, during the course of your work have you been subjected to… threats and humiliating behaviour?
		Intimidation by superiors or colleagues	In the last 12 months, have you been subjected to physical violence in your work place?	Over the past 12 months, have you been threatened … at the workplace in such a way that you felt scared?	What about physical violence such as being held or pushed?		Have you been subject to any of the following during the last twelve months?...physical violence from people outside your workplace	And over the past 12 months, during the course of your work have you been subjected to … physical violence?

Country	Netherlands	Denmark	Norway	Finland	Germany	Spain	Europe
	Physical violence by clients (or patients, students, passengers etc.)		Over the past 12 months, have you been exposed … to violence at the workplace that caused visible marks or physical damage? [yes, no, if yes: How many times?]			Have you been subject to any of the following during the last twelve months?… verbal abuse, rumors or social isolation	And over the past 12 months, during the course of your work have you been subjected to … bullying / harassment?
	Physical violence by superiors or colleagues		Over the past 12 months, have you been exposed … to violence at the workplace that did not cause visible marks or physical damage? [yes, no, if yes: How many times?]				And over the past 12 months, during the course of your work have you been subjected to … sexual harassment?
	Bullying by clients (or patients, students, passengers etc.)						
	Bullying by superiors or colleagues						
**workplace conflict**	Did you (in the last 12 months) have a conflict with: one or more of your direct colleagues?		At your workplace, do you experience bad relations between managers and employees?				
	Did you (in the last 12 months) have a conflict with: your direct superior?		At your workplace, do you experience bad relations between employees?				
	Did you (in the last 12 months) have a conflict with: your employer?		At your workplace, do you find yourself involved in unpleasant conflicts with superiors?				
			At your workplace, do you find yourself involved in unpleasant conflicts with colleagues?				
